# The Standard Error/Standard Deviation Mix-Up: Potential Impacts on Meta-Analyses in Sports Medicine

**DOI:** 10.1007/s40279-023-01989-9

**Published:** 2024-01-25

**Authors:** Gavin Sandercock

**Affiliations:** University of Essex, Wivenhoe Park, Colchester, CO43SQ UK

## Abstract

**Background:**

A recent review found that 45% of meta-analyses included statistical errors, of which, the most common was the calculation of effect sizes based on standard error (SE) rather than standard deviation (SD) [the SE/SD mix-up].

**Objectives:**

The first aim of this study was to assess the impact of the SE/SD mix-up on the results of one highly cited meta-analysis. Our second aim was to identify one potential source of the SE/SD mix-up, by assessing how often SE is reported as a measure of sample variability in randomised controlled trials in sports medicine.

**Methods:**

We checked for potential SE/SD mix-ups in a 2015 meta-analysis of randomised controlled trials reporting the effects of recreational football interventions on aerobic fitness in adults. We corrected effect sizes affected by SE/SD mix-ups and re-analysed the data according to the original methodology. We compared pooled estimates of effect sizes from our re-analysis of corrected values with those of the original study. To assess how often SE was reported instead of SD as a measure of sample variance, we text mined results of randomised controlled trials from seven sports medicine journals and reported the proportion reporting of SE versus SD.

**Results:**

We identified potential SE/SD mix-ups in 9/16 effect sizes included in the meta-analysis describing the effects of football-based interventions versus non-exercise control. The published effect size was standardised mean difference (SMD) = 1.46 (95% confidence interval [CI] 0.91, 2.01). After correcting for SE/SD mix-ups, our re-analysis produced a smaller pooled estimate (SMD = 0.54 [95% CI 0.37, 0.71]). The original pooled estimate for trials comparing football versus running interventions was SMD = 0.68 (95% CI 0.06, 1.4). After correcting for SE/SD mix-ups and re-analysis, the effect was no longer statistically significant (SMD = 0.20 [95% CI − 0.10, 0.49)]). We found that 19.3% of randomised controlled trials reported SE rather than SD to describe sample variability. The relative frequency of the practice ranged from 0 to 25% across the seven journals sampled.

**Conclusions:**

We found the SE/SD mix-up had inflated estimates for the effects of football on aerobic fitness. Meta-analysts should be vigilant to avoid miscalculating effect sizes. Authors, reviewers and editors should avoid and discourage (respectively) the practice of reporting SE as a measure of sample variability in sports medicine research.

**Supplementary Information:**

The online version contains supplementary material available at 10.1007/s40279-023-01989-9.

## Key Points

**Table:** 

We identified multiple standard error/standard deviation (SE/SD) mix-ups in a meta-analysis comparing the effects of football on adult fitness versus non-exercise conditions and running interventions.
Correcting for SE/SD mix-ups reduced pooled effect size estimates for football interventions versus non-exercise controls from standardised mean difference = 1.43 (95% confidence interval 0.79, 2.07) to standardised mean difference = 0.54 (95% confidence interval 0.37, 0.71); formerly significant differences between football and running became non-significant.
To identify potential sources of the SE/SD mix-up, we text mined 3493 randomised controlled trials published in seven sports medicine journals; 19.3% included SE as a measure of sample variability.

## Introduction

A recent study by Kadlec et al. [1] highlighted the prevalence and illustrated the potential impact of common errors found in meta-analyses within the strength and conditioning literature. Inspired by this work, we sought to draw comparisons from within the sports medicine literature, by re-analysing a highly cited meta-analysis as a worked example. We also aimed to investigate whether common errors highlighted by Kadlec et al. could explain the reported efficacy of recreational football as an intervention to improve aerobic fitness ($\dot{V}$O_2max_ mL·kg^−1^·min^−1^).

The example used was the 2015 meta-analysis of randomised controlled trials (RCTs) reporting the effects of recreational football on adults’ aerobic fitness ($\dot{V}$O_2max_, mL·kg^−1^·min^−1^) by Milanovic et al*.* [2]. When compared with non-exercise controls, the effect of recreational football on $\dot{V}$O_2max_ is impressive (standardised mean difference [SMD] = 1.46 (95% confidence interval [CI] 0.91, 2.01). The intermittent bouts of high-intensity activity that characterise football were proposed to explain the $\dot{V}$O_2max_ effect. However, the effect size reported is near double that reported for repeat sprint training interventions [3, 4] and high-intensity interval training [5], and larger than the pooled estimate reported for running training in adults [6] and older adults [7]. This particular study was chosen because of the large effect size reported and because forest plots showed studies with very large effect sizes (SMD > 3), the threshold above which Kadlec et al. [1] suggested readers should exert a high degree of suspicion.

A visual inspection of forest plots of a secondary analysis comparing football versus continuous running also revealed conspicuously large individual effect sizes for two studies relative to the remainder included in the analysis. Both effect sizes were plausible (SMDs ~ 1.5). Evidence for an intervention that can improve aerobic fitness more than continuous running has the potential to impact public health policy and practice, particularly in an already popular and well-funded sport such as football. The overall effect size (SMD = 0.68) appeared plausible, but this finding—that football improves fitness more than continuous running—runs contrary to evidence for repeated-sprint training [3, 4] and high-intensity interval training [5] interventions. We chose to re-examine the analysis comparing football with running to ensure that this claim was based on robust evidence and sound meta-analytical practices.

A modified version of Kadlec et al.’s checklist is shown in Table 1, alongside the actions taken in applying the checklist and a brief justification of each. Milanovic et al. [2] included only data from RCTs of recreational football. The methods state that studies were appropriately weighted (random effects models). The outcome is clearly defined as aerobic fitness ($\dot{V}$O_2max_ mL·kg^−1^·min^−1^), this single value has few surrogates and none appear to have been reported. These factors negated the need to fully assess items 1–3 on the checklist.

**Table 1:** Modified version on Kadlec et al.’s [1] checklist for errors in a meta-analysis as applied in this present study

	Checklist item	Action taken and justification
1	Focus on within-group comparison	Not applicable: meta-analysis includes only randomised controlled trials
2	Fail to account for correlated observation	Not assessed: typically, only one comparison of $\dot{V}$O_2max_ values in each separate analysis
3	Failure to weight studies	Studies included in the analysis were appropriately weighted using random effects models (inverse variance method)
4	Outliers (SMD > 3.0)	Assessed by adopting the recommended cut-off for the effect size: > 3.0
5	SE/SD Mix-up	Assessed, initially to explain outliers; eventually assessed for all included studies by accessing the full text of original papers

*SD* standard deviation, *SE* standard error, *SMD* standardised mean difference

Instead, this study focuses on factors relating to errors 4 and 5 in Table 1 [1], the inclusion of undetected outliers, the cause of outlying values and their influence on results. When defined as an SMD > 3.0, potential outliers are relatively easy to identify if forest plots are reported, but determining whether an outlier is erroneous, and the cause of these errors is more complex. Kadlec et al. [1] found 60% of outliers could be attributed to the use of standard error (SE), rather than standard deviation (SD) as the denominator when calculating effect sizes; they termed this the ‘SE/SD mix-up’. In their re-analysis of Seitz et al. [8] correcting just one value, Kadlec et al*.* [1] adeptly illustrated how the SE/SD mix-up can inflate pooled estimates of the meta-analysis.

Our first aim was to determine of the presence of SD/SE mix-ups and to quantify their effects on a pooled effect size estimates of a highly cited meta-analysis in sports medicine. To do this, we first replicated the original analysis as described. We then checked and corrected any potential SE/SD mix-ups and re-analysed the data. Differences in individual study effect sizes were taken as evidence of SE/SD mix-ups. Differences in the pooled estimates of effect sizes were used to illustrate the effects that these mix-ups had on the original study results.

The confusion over the use of SE and SD has been the subject of research, [9–14] debate [9] and the topic of multiple educational articles [15–19]. Reporting of SE instead of SD to describe sample variability was listed as one of the 20 most common mistakes in biomedical research [20]. The reporting of SE rather than SD when describing sample variability appears commonplace across a number of scientific disciplines [11, 13, 16, 20, 21]. Our second aim was to investigate how commonly SE, rather than SD, is reported as a measure of sample variability in the sports medicine literature.

## Methods

### SE/SD Mix-Up

There is no single way of identifying outliers in meta-analyses as they are dependent on the context. Commonly used rules of thumb are values that are more than > 3 SD from the mean or more than 1.5 times the interquartile range from the median. Kadlec et al. [1] noted that many studies included in the meta-analyses they reviewed had “*surprisingly small SDs*” but that it was unclear if these potential outliers were miscalculations. Ideally, any potential outliers need to be evaluated on an individual basis. Therefore, regardless of the effect size, we accessed the full-text versions of each study included in the meta-analysis of Milanovic et al. [2], and extracted the means and measures of sample variability as reported. For each effect size, we recorded whether the statistic accompanying each mean was clearly reported and whether this was SD or SE.

### Replication of the Original Analysis

Replication was problematic as did not report the values used to calculate effect sizes. To verify SD or SE as the denominator, we instead had to manually re-extract means, and ‘denominator’ values as reported in the original studies. We then replicated based on the original methodology to verify whether we had extracted the same values used in the original study. Successful replication of study effect sizes allowed us to identify potential SE/SD mix-ups, before correcting them to undertake our re-analysis.

Replication was difficult because of omissions in the original methodology. For instance, the authors did not explicitly state which values were extracted from studies in order to calculate SMD. As all included studies were RCTs, with two (or more) independent groups we assumed SMDs were calculated based on independent post-test group values. The natural unit of difference is that calculated from between the post-test means. The standardiser used to calculate SMD normally derived from the pooled SDs of the independent groups (see Eq. 1).

Calculation of SMD:

1 $${\text{SMD}} = \frac{{\mu 1 {-} \mu 2}}{{\sqrt {\left( {\left( {\sigma 12 + \sigma 22} \right)/2} \right)} }},$$

where *μ*1 is the post-test mean of the intervention group, *μ2* is the post-test mean of the control group, *σ*1 is the post-test SD of the intervention group, and *σ*2 is the post-test SD of the control group.

Milanovic et al. [2] stated: *“The standardized mean differences and 95% confidence intervals (CIs) were calculated for the included studies”.* We assumed the authors calculated Cohen’s ‘*d*’ as this appears to be the default in the software (Comprehensive Meta-Analysis, Version 3; Biostat, Englewood, NJ, USA) used by the authors [2]. Based on the sample sizes of the included studies (median *n* = 15, range *n* = 7–34), we also calculated SMD using Hedge’s ‘*g*’ correction for small samples.

We replicated two analyses from the original study [2]; ‘Recreational football versus non-exercise controls’ and ‘Recreational football versus running’. Three other analyses were originally reported (‘All studies’, ‘Males’ and ‘Females’) but each contained duplicate effects for the same ‘football group’ from individual studies (e.g. ‘Football versus control’ and ‘Football versus exercise’). The analyses also duplicated one another (Males and Females both being subsets of ‘All Studies’).

The original study methods state only that *‘random effects models’* were used. For replication, we therefore also used random effects models (restricted maximum likelihood) to calculate pooled estimates of SMD (as Hedge’s *g*). All analyses were carried out using the Meta-Analysis application in JASP, Version 0.17.3 (https://jasp-stats.org/). This approach closely replicated the original findings. Replication was needed to ensure a valid comparison of results based on uncorrected and corrected values.

Where original studies reported SE, rather than SD as a measure of sample variability, we converted SE to SD (by multiplying by the square root of *n*) and re-calculated the effect sizes and re-ran the meta-analyses. By comparing the pooled estimates of effect sizes between uncorrected and corrected analyses, we were able to quantify the influence of SE/SD mix-ups on the results of the original study.

### Prospective Comparison of Original Effect Sizes with Values from the Re-Analysis

To examine whether the estimates of effect sizes from our re-analysis were realistic, we updated the original analysis. We included studies on the effects of football interventions on $\dot{V}$O_2max_ of adults that included a non-exercise control condition. Full search criteria, extraction and a PRISMA (Preferred Reporting Items for Systematic Reviews and Meta-Analyses) flow diagram are supplied as Electronic Supplementary Material (ESM). Using a random effects model (restricted maximum likelihood), we calculated pooled estimates of effect size (Hedge’s g). We compared both the original study estimates and those from our re-analysis with the pooled estimate obtained from studies published since the original meta-analysis [2].

### Practice of Reporting SE as a Measure of Sample Variability

We used Orange 3.27 for Windows (https://orangedatamining.com/), which includes a PubMed text mining application and Text Analytics Widgets to search for RCTs published in seven Q1 sports medicine journals indexed in PubMed. We pre-processed text by applying lowercase transformation then tokenised text at a ‘word space’ level. The processed text was mined for SE values using the Concordance Widget using four separate terms ‘se’, ‘standard error’, ‘sem’ and ‘standard error of the mean’. We mined the same text for SDs using two terms ‘sd’ and ‘standard deviation’. Each search term returned ten-word concordances plus study index numbers that were exported to combined data tables where we removed any duplicate concordances drawn from individual studies.

We identified SE/SD mix-ups where concordances contained expressions such as ‘mean (SE =) or ‘mean (± SE)’ to describe sample variability (often as descriptive statistics) or other uses associated with non-inferential analyses. Studies reporting SE to describe the results of inferential analyses (mean change, mean difference or regression analysis) were identified automatically if concordances included symbols or words associated with reporting inferential analyses (*β* = , *F* = , ANOVA, mean difference) and deemed as correct use as were studies containing ‘mean ± SD’ or mean (SD). The number of correct use studies was used as the comparator to estimate the relative frequency of SE/SD mix-ups in each journal. A full list of search terms, all studies assessed and their classifications are available as an Excel file in the ESM.

## Results

### Inclusion of Undetected Outliers

We identified three potential undetected outliers studies with effect sizes ~ 3 in the analysis comparing football interventions with non-exercise control conditions. Manual checks confirmed that all three studies [21–24] reported descriptive statistics as mean and SE. Reporting of SE was clearly stated within the results of each study, including the legends of figures or tables.

### Effects of Football Interventions Versus Non-Exercise Control Conditions

**Fig. 1:**
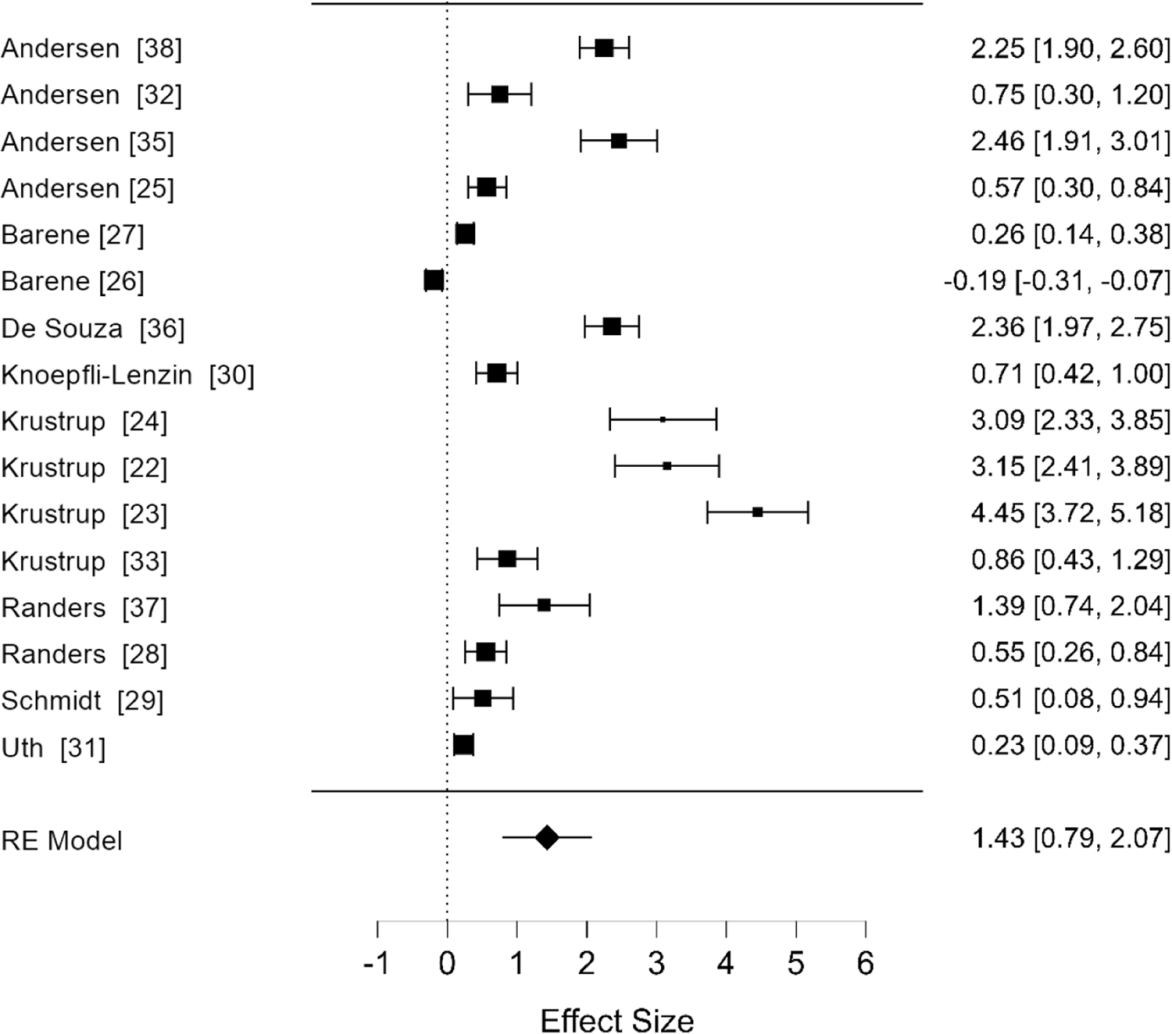
Replication of the original analysis including original effect sizes. Forest plot comparing effects of recreational football versus non-exercise control conditions on $\dot{V}$O_2max_ in adults. *RE* random effects

Our replication of the analysis comparing the effects of recreational football with non-exercise control conditions is shown in Fig. 1. This figure shows uncorrected values (SDs and SEs as effect size denominators) and therefore includes the three outlying values discussed above.

The replication analysis pooled estimate was SMD = 1.43 (95% CI 0.79, 2.07) with a high degree of between-study heterogeneity (*I*^2^ = 90.4%). The original analysis reported a pooled estimate of SMD = 1.46 (95% CI 0.91, 2.01) with high heterogeneity (*I*^2^ = 88.4). These similarities strongly suggested successful replication and that the original analysis included multiple miscalculated effect sizes in addition to the three outliers [21–24].

Of the 16 studies included, seven reported $\dot{V}$O_2max_ values as mean and SD [24–31]. The remaining nine reported mean plus SE as a measure of sample variability (either in tables of descriptive statistics or in results [21–24, 31–38]). Standard error was most commonly reported as SEM but in all cases the statistic reported was clearly stated. When we converted SE values to SD, recalculated the effect sizes and repeated the analysis, the pooled estimate was SMD = 0.54 (95% CI 0.37, 0.71) and there was a modest reduction in heterogeneity (*I*^*2*^ = 78.4%).

### Recreational Football Versus Running Interventions

**Fig. 2:**
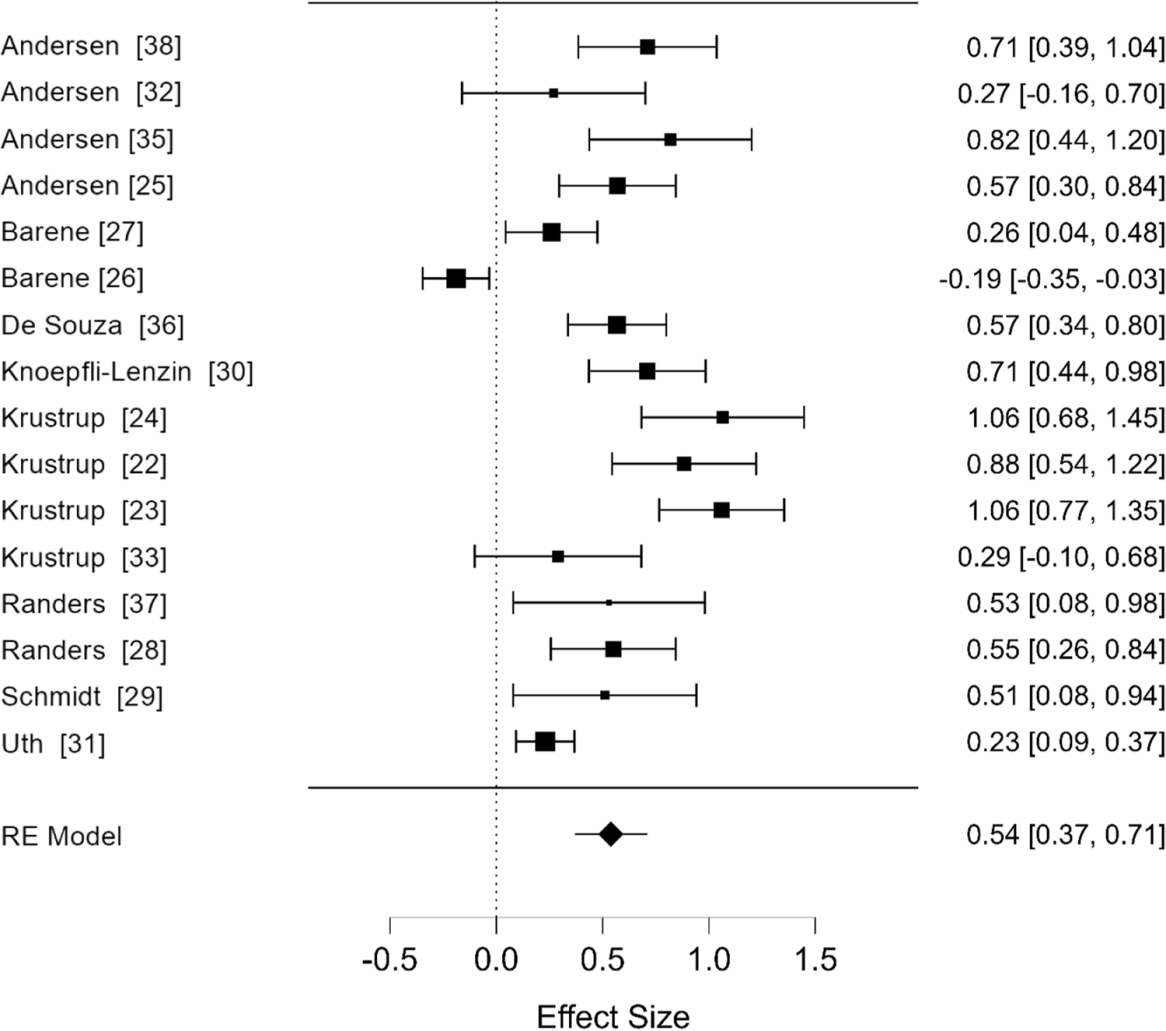
Re-analysis after correction of miscalculated effect sizes. Forest plot of studies comparing the effects of recreational football versus non-exercise control conditions on $\dot{V}$O_2max_ in adults. *RE* random effects. (Note that the *x-axis* showing the effect size has been rescaled from − 1.0 to 6.0 to − 0.5 to 1.5)

The uncorrected analysis included six studies that compared the effects of recreational football on adults’ $\dot{V}$O_2max_ compared with recreational running. A replication of the original analysis based on uncorrected values produced a pooled estimate of SMD = 0.68 (95% CI 0.06, 1.30). This close approximation confirmed successful replication of the original study (SMD = 0.68 (95% CI 0.07, 1.29)) and that the original study included effect sizes based on the SE/SD mix-up. We found that four of the six original studies reported SE, rather than SD (Fig. 2).

**Fig. 3:**
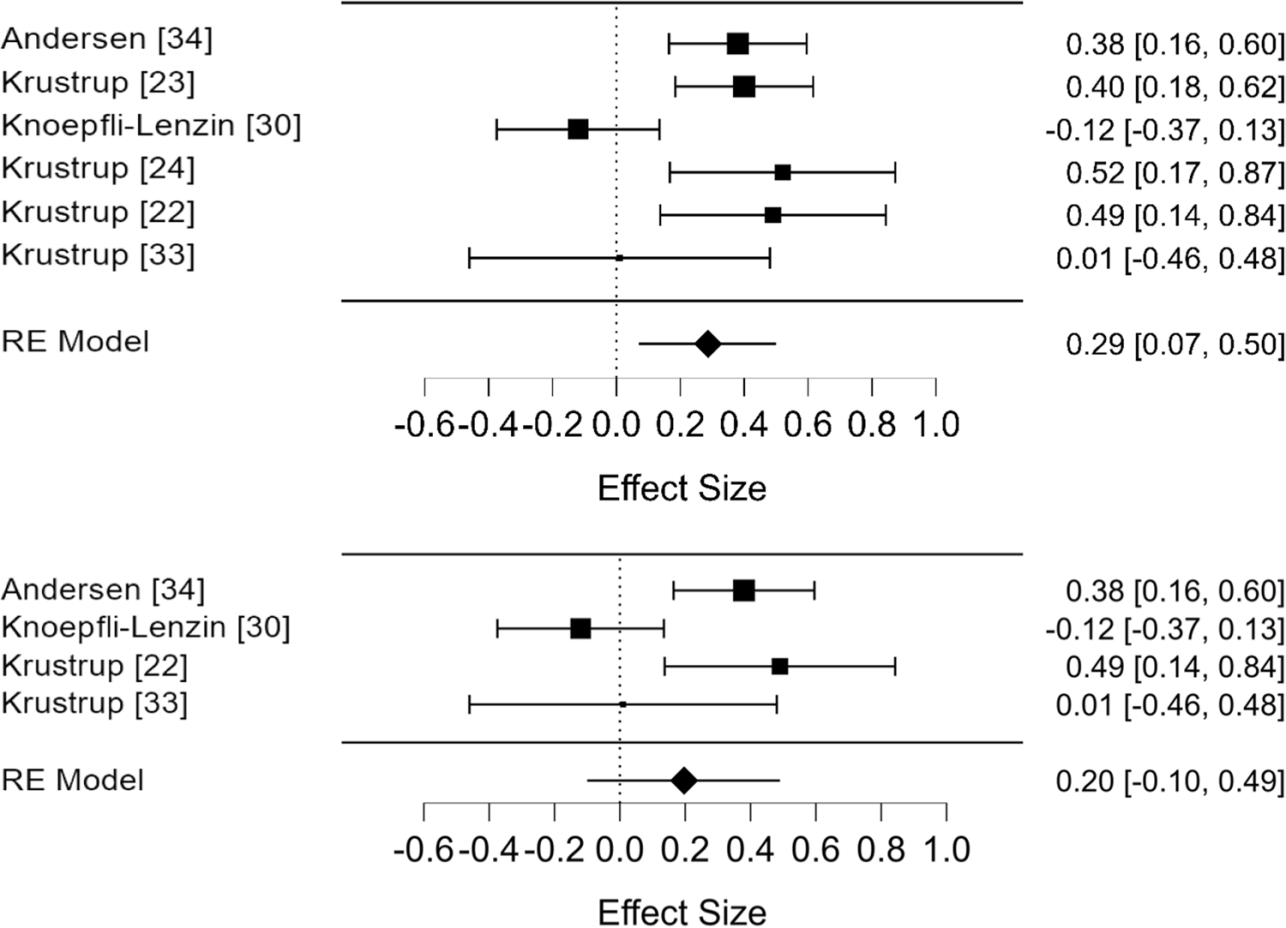
Effects of recreational football versus running on $\dot{V}$O_2max_ in adults. **a** Re-analysis including four effect sizes after correcting for the standard error/standard deviation mix-up and **b** Re-analysis after correcting for the standard error/standard deviation mix-up and after removal of two suspected duplicates. Potential duplicate pairs are: [22, 24] and [33, 34]. *RE* random effects

A re-analysis using corrected effect sizes (Fig. 3a) initially suggested football was more effective than running although the pooled estimate was small SMD = 0.29 (95% CI 0.07, 0.50). Following correction of the SE/SD mix-up (Fig. 3a), we noticed two studies with near-identical effect sizes [22, 24], suggesting a possible duplication. Study data differed due to one additional participant being initially included in the football group. Identical values for $\dot{V}$O_2max_ at baseline and follow-up were reported for the running group. The very similar effect sizes for two further studies, this time in women [23, 34] also appear to be duplications. The running group had identical baseline values for $\dot{V}$O_2max_ in both studies. The slight variation in effect size again seems to be due to one additional participant being included in the football group in one of the studies [33]. When we re-analysed the data excluding these possible duplicates, the effect size was non-significant at SMD = 0.20 (95% CI − 0.10, 0.49) and heterogeneous (*I*^2^ = 77.1%).

## Discussion

Kadlec et al. [1] noted that about 60% of all effect sizes > 3.0 were due to the ‘SE/SD error’. All three effect sizes > 3.0 were attributable to the SE/SD mix-up and indicate that effect sizes around 3.0 should have a high index of suspicion for error. As effect sizes from within-group differences tend to be larger between-group comparisons, it may even be prudent to lower this threshold (or apply it pragmatically) for meta-analyses of RCTs.

All outliers were attributable to the SE/SD mix-up [22, 23] but not every SE/SD mix-up created an outlier. The majority of SE/SD mix-ups were harder to spot [1] because, while incorrect, the resultant effect sizes were plausible. Because the original study did not provide the values used to calculate effect sizes, we had to manually check the original version of each study in order to identify SE/SD mix-ups. We strongly encourage all authors and reviewers to insist that the data extracted from studies and used to calculate effect sizes be included in published meta-analyses.

Importantly, all studies clearly stated whether SE/SEM or SD was reported. Where reported, SE values were easily identified. Authors of prior meta-analyses of sports-based [39] and running-based [6] interventions have included some of the same studies reporting SE. Like them, we were able to convert SE to SD before calculating effect sizes. This approach is not practical for the readers of meta-analyses and was only necessary for the present study because of the unusually high prevalence of SE/SD errors in the original analysis.

Kadlec et al. [1] reported that most of the highly cited meta-analyses they reviewed included at least one incorrectly calculated effect size, but did not assess the actual prevalence within individual meta-analyses. We found that 56% (9/16) of the effect sizes meta-analysed in the comparison of football interventions versus non-exercise controls were incorrectly calculated. All miscalculations were due to the SE/SD mix-up. Given the impact the SE/SD mix-up had on the results after correction and re-analysis, we sought to identify potential sources of the error.

In the present study, we found, however, that 66% of SE/SD mix-ups came from the studies published in the same journal [22, 23, 33, 34–37]. As of January 2023, the *Scandinavian Journal of Medicine and Science in Sports* instructions to authors regarding data tables state: ‘*Statistical measures such as SD or SEM should be identified in the headings*.’ We suggest that some authors may interpret this instruction as a ‘choice’ between ‘SD or SEM’ as the instruction could be perceived as inferring they are interchangeable.

The SD is a measure of sample variability and provides an estimate of population variability. Equation (2) shows that the numerator is derived from ${{\text{x}}}_{{\text{i}}}$ (each value within the data set). The numerator (*N*) represents the total number of values in the data set. As such, SD is not affected by sample size.

Calculation of SD ($\sigma$):

2 $$\sigma =\frac{\sqrt{{\sum ({x}_{i}-\mu )}^{2}}}{N},$$

where *x*_*i*_ is each value in the data set, $\mu$ is the population mean and *N* is the total amount of values in the data set.

The SE is an estimate of the precision of the sample mean (Eq. 3). While SD is not affected *N*, SE decreases in proportion to the sample size.

SE/SEM

3 $$\mathrm{SE }=\frac{\sigma }{\sqrt{N}},$$

where *σ* is the standard deviation and *N* is the total amount of values in the data set.

### How Common is the Practice of Reporting of SE as a Measure of Sample Variability?

We used text mining to determine whether this particular journal included higher-than-expected reporting of SE as a measure of sample variability and to investigate how common this practice is within the sports medicine literature. We searched seven sports medicine journals listed in PubMed by name, using the PubMed type of study filter ‘Randomized Controlled Trial’. The initial search returned the total number of RCTs published in each journal. Using these studies as our Corpus, we used a text mining analysis based on simple text analytics (concordance analysis) to identify all studies reporting either SD or SE within the text of the results section. To account for differences in the overall number of RCTs published and in the proportion that reported SD or SE, we randomly re-sampled available values so that the final sample represented ~ 10% of RCTs published in each journal. The proportion of RCTs returned that reported either SD or SE and the proportion included in the analysis is provided in Table 1 of the ESM.

The prevalence of reporting SE to describe sample variability across all seven journals sampled was 19.3%; above the 13% reported for obstetrics and gynaecology research [10] similar to the 23% rate reported for anaesthesia journals [11] but much lower than in cardiovascular journals [14]. While prevalent, the causes of such this practice cannot be determined here. The practice could simply stem from naivety and a misunderstanding of when SE should (and should not) be used [15, 17]. It has, however, been suggested that authors may choose to report SE (in place of SD) as a deliberate ploy to make data appear less variable [13] or to make figures more visually appealing with smaller error bars [12]. There is evidence in biomedical research that studies including ‘*impressive-looking’* findings (substituting SE for SD) are cited more often than those reporting SD [10, 17].

Regardless of how often authors report SE values as measures of sample variability, responsibility for the accuracy of any meta-analysis still lies, ultimately, with the authors and reviewers. The authors of the meta-analysis in question stated: *“In most of the studies, mean and standard deviation (SD) pre- and post-values were reported.”* This statement is untrue (SE was reported more often than SD) in the included studies. Where SE was reported, this was clearly stated, and we were able to identify all incidences where this occurred. Authors of meta-analyses that include some of the same studies were also able to identify and correct SE values reported [6, 39].

### What Does this Mean for the Evidence for Recreational Football?

When Kadlec et al. [1] corrected and re-analysed the meta-analysis of Seitz et al. [8], the results still supported the original conclusions, albeit with a smaller effect size (weaker evidence). The high prevalence of errors found in the evidence for football’s effects on aerobic fitness had a more pronounced (downward) shift in summary effects.

Compared with non-exercise control conditions, the results of our corrected re-analysis do still support the original conclusion that football is better than non-exercise conditions. Rather than the impressive effect size reported originally (SMD = 1.46 [95% CI 0.91, 2.01]), our analysis suggests recreational football has a medium effect on $\dot{V}$O_2max_ (SMD = 0.54; [95% CI 0.37, 0.71]) more in agreement with those reported for repeated-sprint training (SMD = 0.63 [95% CI 0.39, 0.87]) [3] and structured high-intensity interval training interventions (SMD = 0.69 ([95% CI 0.46, 0.93]) [40].

The corrected data still support the original conclusion—that recreational football improves aerobic fitness when compared with non-exercise conditions. More contentious was the authors’ original finding that recreational football was a more effective way to improve aerobic fitness than running. We found that four of the six effect sizes in the analysis had been incorrectly calculated using SE. After correction, there was still evidence for a small benefit of football over running (SMD = 0.29 [95% CI 0.07, 0.50]). Reporting corrected effect sizes in forest plots (Fig. 3b) suggested the possible inclusion of duplicate values. A full discussion on the inclusion, detection and influence of duplicates is beyond our stated scope, but removing the two suspected duplicates had an important effect on the results that challenged the overall conclusion. The overall effect size (SMD = 0.20 [95% CI − 0.10, 0.49]) was non-significant; the results no longer supported the original conclusion that playing recreational football is significantly better at improving adult fitness compared with running interventions.

### Meta-Analysis of Football-Based Interventions Published Since 2015

**Fig. 4:**
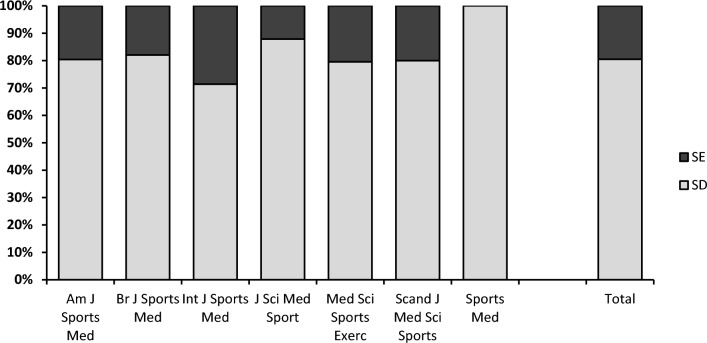
Reporting of standard error (SE) versus standard deviation (SD) as a measure of sample variability in the results of randomised controlled trials published in seven sports medicine journals. For each journal, the results are shown for a sample representing 10% of randomised controlled trials: Am J Sports Med, *n* = 51; Br J Sports Med, *n* = 39; Int J Sports Med, *n* = 49; J Sci Med Sports, *n* = 33; Med Sci Sports Exerc, *n* = 124; Scand J Med Sci Sports, *n* = 50, Sports Med, *n* = 15

Estimates from meta-analyses are often cited in sample size calculations, but inflated effect sizes will artificially reduce estimates for sample size estimates, leading to underpowered studies. If summary estimates of effect size are artificially inflated, they are unlikely to be replicated in consequent studies (Fig. 4).

**Fig. 5:**
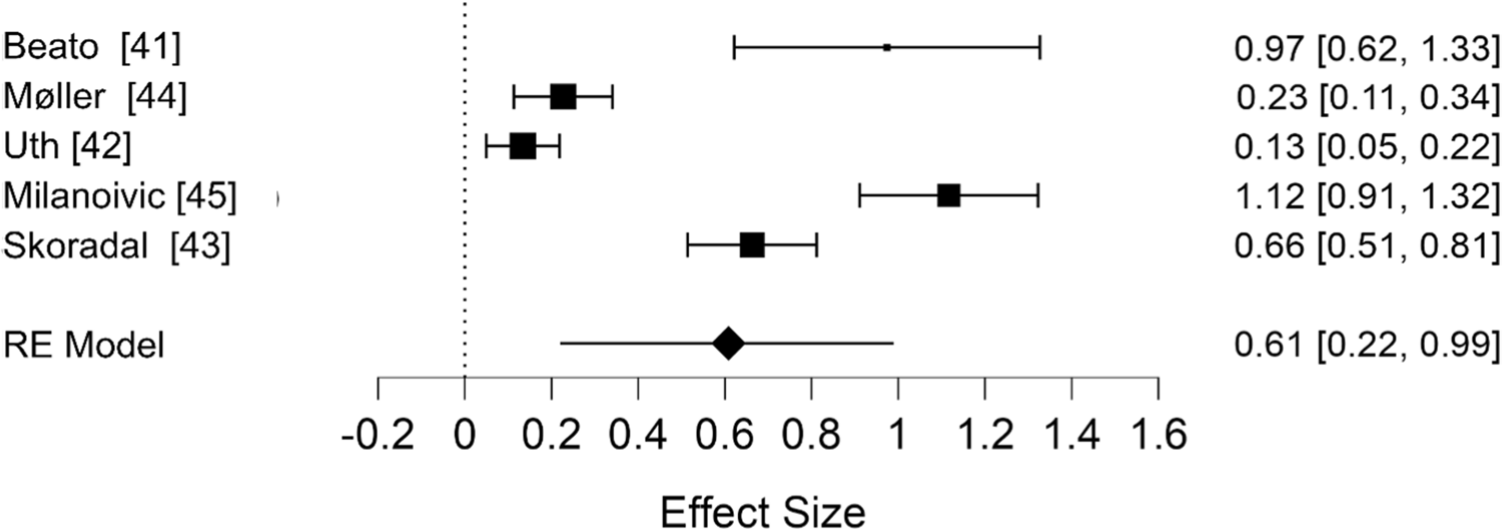
Updated meta-analysis of the effectiveness of randomised controlled trials of recreational football versus non-exercise controls (published since 2015). *RE* random effects

To test this hypothesis, we identified studies reporting the effects of football on aerobic fitness versus non-exercise controls based on the inclusion criteria listed in the original study [2]. The search returned five additional studies [40–45]. Again, we meta-analysed these data, using a random effects model (restricted maximum likelihood) to calculate pooled estimates of SMD (expressed as Hedge’s g). The summary effect size of the five studies included (Fig. 5) was medium (SMD = 0.61 [95% CI 0.22, 0.99]) and heterogeneous (*I*^2^ = 88.5%). The estimates from these studies were close approximations of our re-analysis of corrected values, rather than the original findings as reported.

## Conclusions

The presence of SE/SD mix-ups explained all the undetected outlying values in the example meta-analysis. The calcualtion of effect sizes using SE instead of SD effects had a major impact on the results of the meta-analysis, reducing the overall estimate nearly three-fold. While the overall conclusions that football is beneficial to fitness remain supported by the results, the corrected magnitude of this effect is much smaller, but also in agreement with comparable exercise interventions. After correcting calculation errors and removing duplicate values, the conclusions regarding football versus running were no longer supported by the results.

Readers of meta-analyses should be aware of the prevalence of the miscalculation of effect sizes and the inflationary influence they have on pooled estimates. We suggest checking any outlying values obvious to the eye in all meta-analyses before assuming correctness. Readers are advised to routinely check the largest effects present in any meta-analysis.

Authors, reviewers and editors should take steps to ensure that SE is not used in place of SD in empirical studies. The absence of SE/SD mix-ups in one journal ‘*Sports Medicine*’, which has an explicit editorial policy on the matter, suggests that the goal of eliminating SE/SD mix-ups is achievable.

### Supplementary Information

Below is the link to the electronic supplementary material.

Supplementary file1 (DOCX 65 KB)

Supplementary file2 (DOCX 25 KB)

Supplementary file3 (XLSX 12 KB)

## Declarations

### Availability of data and material

All data used in this study are available as supplementary materials.
