# Supplementary material for: The Standard Error/Standard Deviation Mix-Up: Potential Impacts on Meta-Analyses in Sports Medicine
Source: Sports Med. 2024 Jan 25;54(6):1723–32. doi: 10.1007/s40279-023-01989-9 (PMC11239727; doi:10.1007/s40279-023-01989-9)
Supplement: Supplementary file 1 — Supplementary file1 (DOCX 65 KB) [file 40279_2023_1989_MOESM1_ESM.docx]

Supplementary Materials: Methods

Prisma Flow Diagram or: An updated meta-analysis of the effectiveness of randomized controlled trials (published after 2015) of recreational football versus non-exercise controls

**Identification of studies via databases and registers**

Records removed *before screening*:

Duplicate records removed (n = 47)

Records marked as ineligible by automation tools (n = 0)

Records removed for other reasons (n = 7)

Records identified from*:

PubMed (n =49)

Web of Science (n = 55)

**Identification**

Records screened.

(n = 50)

Records excluded**

(n = 12)

Reports sought for retrieval

(n = 38)

Reports not retrieved

(n = 0)

**Screening**

Reports assessed for eligibility

(n = 38)

Reports excluded:

Participants <19 years (n = 9)

Elite or Professional (n = 19)

No measure of Vo2max (n=4)

No non-exercise control group (n = 1)

Studies included in review.

(n = 5)

Reports of included studies

(n = )

**Included**

Search Terms: (((football) OR (soccer)) AND (fitness) AND (("2015"[Date - Publication] : "2023"[Date - Publication])) AND (randomizedcontrolledtrial[Filter]))

*From:*  Page MJ, McKenzie JE, Bossuyt PM, Boutron I, Hoffmann TC, Mulrow CD, et al. The PRISMA 2020 statement: an updated guideline for reporting systematic reviews. BMJ 2021;372:n71. doi: 10.1136/bmj.n71

For more information, visit: <http://www.prisma-statement.org/>

Supplementary Materials:

Flow Diagram showing identification retrieval and inclusion of studies used in data mining to investigate the reporting of standard error as a measure of sample variability in the sports medicine literatue.

**Identification of Studies for Text Mining**

Records identified:

PubMed (n = 3493)

Am J Sports Med (n = 479)

Br J Sports Med (n = 361)

Int J Sports Med, (n = 470)

J Sci Med Sports (n = 333)

Med Sci Sports Exerc (n = 1230)

Scand J Med Sci Sports (n = 480)

Sports Med (n = 140)

**Identification**

**Reports excluded:

SD or SE/SEM not identified in text:

Am J Sports Med (n = 428)

Br J Sports Med (n = 322)

Int J Sports Med (n = 421)

J Sci Med Sports (n = 300)

Med Sci Sports Exerc (n = 988)

Scand J Med Sci Sports (n = 430)

Sports Med (n = 99)

Reports text mined and assessed for eligibility based on extraction of: SD or SE/SEM (n = 3493)

**Screening**

Am J Sports Med (n = 51)

Br J Sports Med (n = 39)

Int J Sports Med, (n = 49)

J Sci Med Sports (n = 33)

Med Sci Sports Exerc (n =132)

Scand J Med Sci Sports (n = 50)

Sports Med (n = 40)

**Mined**

Am J Sports Med (n = 47)

Br J Sports Med (n = 37)

Int J Sports Med, (n = 48)

J Sci Med Sports (n = 33)

Med Sci Sports Exerc (n = 124)

Scand J Med Sci Sports (n = 49)

Sports Med (n =14)

**Included**

#All Sports Medicine Journals are indexed in Pubmed. Original search terms: ("Medicine and science in sports and exercise"[Journal] OR "The American journal of sports medicine"[Journal] OR "International journal of sports medicine"[Journal] OR "Scandinavian journal of medicine science in sports"[Journal] OR "Journal of science and medicine in sport"[Journal] OR "British journal of sports medicine"[Journal]) OR "Sports medicine"[Journal]) AND (randomizedcontrolledtrial[Filter])#All Sports Medicine

**Excluded by automated text mining app as concordance analyses were unable to identify required terms within article text.
