# Supplementary material for: The Standard Error/Standard Deviation Mix-Up: Potential Impacts on Meta-Analyses in Sports Medicine
Source: Sports Med. 2024 Jan 25;54(6):1723–32. doi: 10.1007/s40279-023-01989-9 (PMC11239727; doi:10.1007/s40279-023-01989-9)
Supplement: Supplementary file 2 — Supplementary file2 (DOCX 25 KB) [file 40279_2023_1989_MOESM2_ESM.docx]

**Supplementary Materials**

**Possible Duplicates.**

The authors give no indication of how they identified or dealt with outliers and only state that duplicates were reviewed; providing no details of how many were identified or reviewed. Using the corrected values, we compared effect size values between studies and manually extracted original ethical approval IDs and baseline values for V̇O_2max_ of the intervention and control groups to identify potential duplicate values within the meta-analysis.

**Table S1.** Duplicate values reported for change in V̇O_2max_ in men receiving recreational football versus running interventions.

|  | **Krustrup (2010)** | **Krustrup (2009)** |  |
| --- | --- | --- | --- |
|  | Muscle adaptations and performance enhancements of soccer training for untrained men | Recreational soccer is an effective health-promoting activity for untrained men | Comparison of Studies & Comments |
| Ethics ID | 14606; H-C-2007-0012 | 14606; H-C-2007-0012 |  |
| Sample | 38 healthy untrained men | 36 healthy untrained men |  |
| Age | 20–43 years | 20–43 years | Identical |
| VO_2max_ (ml min^−1^ kg^−1^) | 39.4 (0.9) | 39.4 (0.9) | Identical |
| Football Group | n=12 | n=12 |  |
| W0 VO_2max_ (ml min^−1^ kg^−1^) | 39.6 (1.5) | 39.6 (1.5) | Identical |
| W4 VO_2max_ (ml min^−1^ kg^−1^) | 42.2 (1.0) | 42.2 (1.0) | Identical |
| W12 VO_2max_ (ml min^−1^ kg^−1^) | 44.5 (1.1) | 44.6 (1.1) | Rounding error |
| Running Group | n=10 | n=10 |  |
| W0 VO_2max_ (ml min^−1^ kg^−1^) | 39.3 (2.5) | 39.3 (2.1) | Identical |
| W4 VO_2max_ (ml min^−1^ kg^−1^) | 41.4 (2.1) | 41.6 (2.0) | Rounding error |
| W12 VO_2max_ (ml min^−1^ kg^−1^) | 42.0 (1.9) | 42.2 (1.8) | High Similarity |
| Control Group | n=10 | n=10 |  |
| W0 VO_2max_ (ml min^−1^ kg^−1^) | 39.2 (2.7) | 39.2 (2.7) | Identical |
| W12 VO_2max_ (ml min^−1^ kg^−1^) | 38.9 (2.4) | 38.9 (2.4) | Identical |

Supplementary table 1 illustrates provides a comparison of key features of two publications included in Milanovic’s meta-analysis. studies similarity and potential duplicate results included in the meta-analysis. Both come from the same research project (Identical ethical approval code). Both assessed participants at 0, 4 and 12 weeks. The only notable difference between the descriptive information is the initial sample size (N=36 versus N=38) although the methods of both studies state that 36 participants were randomized.

Of the eight mean (SE) values describing V̇O_2max_ for the three groups (Football, Running and Control)) five are identical in both studies.

**Table S2**. Duplicate values reported for change in V̇O_2max_ in women receiving recreational football versus running interventions.

|  | **Andersen (2010)** Improvement of systolic and diastolic heart function after physical training in sedentary women | **Krustrup (**2010**)** Beneficial effects of recreational football on the cardiovascular risk profile in untrained premenopausal women | **Comparison of Studies & Comments** |
| --- | --- | --- | --- |
| Ethics ID | 14606;H-C-2007-0012 | 14606;H-C-2007-0012 |  |
| Sample | Healthy sedentary women | Healthy untrained women |  |
| Number | N=47 | N=65 | Subsample |
| Age | 36.5 (8.2) years | 19–47 years |  |
| VO2max (ml min^−1^ kg^−1^) | 34.7 (5.0) | 34.8 (5.4) | High similarity |
| Football Group | *n=18* | *n=19* |  |
| W0 VO_2max_ (ml min^−1^ kg^−1^) | 32.5 (0.9) | 32.7 (1.0) | High similarity |
| W16 VO_2max_(ml min^−1^ kg^−1^) | 37.5 (1.0) | 37.7 (1.0) | High similarity |
| Running Group | n=18 | n=18 |  |
| W0 VO_2max_ (ml min^−1^ kg^−1^) | 35.5 (1.4) | 35.5 (1.4) | Identical |
| W16 VO_2max_ (ml min^−1^ kg^−1^) | 39.1 (1.3) | 39.1 (1.3) | Identical |

Supplementary Table 2 illustrates provides a comparison of key features of two separate studies both of which were included in milanovic’s meta-analysis. [5] [6] studies similarity and potential duplicate results included in the meta-analysis. Both come from the same research project (Identical ethical approval code).

Both assessed participants at 0 and 16 weeks. The only notable difference between the descriptive information is the initial sample size (*N=*65 versus *N=*45) which is because Andersen et al. only compared football (n=18) and running (n=18) groups; whereas Krustrup [5] compared football (n=19) with both running (n=18) and a non-exercise control group (data not shown).

Baseline and follow-up means and SDs for V̇O_2max_ were identical for the running group. There are very small (<1%) differences in the mean VO_2max_ values of the football group – likely due to the presence of an additonal participant. [5].
